# The effect of black cohosh extract and risedronate coadministration on bone health in an ovariectomized rat model

**DOI:** 10.3389/fphar.2024.1365151

**Published:** 2024-04-16

**Authors:** Amy L. Inselman, Elysia A. Masters, Jalina N. Moore, Rajiv Agarwal, Audrey Gassman, Gemma Kuijpers, Richard D. Beger, Kenneth B. Delclos, Sybil Swift, Luísa Camacho, Michelle M. Vanlandingham, Daniel Sloper, Noriko Nakamura, Gonçalo Gamboa da Costa, Kellie Woodling, Matthew Bryant, Raul Trbojevich, Qiangen Wu, Florence McLellen, Donna Christner

**Affiliations:** ^1^ Division of Systems Biology, National Center for Toxicological Research, U.S. Food and Drug Administration, Jefferson, AR, United States; ^2^ Office of New Drug Products, Office of Pharmaceutical Quality, Center for Drug Evaluation and Research, U.S. Food and Drug Administration, Silver Spring, MD, United States; ^3^ Division of Urology, Obstetrics and Gynecology, Center for Drug Evaluation and Research, U.S. Food and Drug Administration, Silver Spring, MD, United States; ^4^ Division of Biochemical Toxicology, National Center for Toxicological Research, U.S. Food and Drug Administration, Jefferson, AR, United States; ^5^ Office of Dietary Supplement Program, Center for Food Safety and Nutrition, U.S. Food and Drug Administration, College Park, MD, United States; ^6^ Office of the Center Director, National Center for Toxicological Research, U.S. Food and Drug Administration, Jefferson, AR, United States; ^7^ Office of Scientific Coordination, National Center for Toxicological Research, U.S. Food and Drug Administration, Jefferson, AR, United States

**Keywords:** black cohosh, risedronate, bone mineral density, dietary supplements, bisphosphonates, postmenopausal osteoporosis

## Abstract

Preparations of black cohosh extract are sold as dietary supplements marketed to relieve the vasomotor symptoms of menopause, and some studies suggest it may protect against postmenopausal bone loss. Postmenopausal women are also frequently prescribed bisphosphonates, such as risedronate, to prevent osteoporotic bone loss. However, the pharmacodynamic interactions between these compounds when taken together is not known. To investigate possible interactions, 6-month-old, female Sprague-Dawley rats underwent bilateral ovariectomy or sham surgery and were treated for 24 weeks with either vehicle, ethinyl estradiol, risedronate, black cohosh extract or coadministration of risedronate and black cohosh extract, at low or high doses. Bone mineral density (BMD) of the femur, tibia, and lumbar vertebrae was then measured by dual-energy X-ray absorptiometry (DEXA) at weeks 0, 8, 16, and 24. A high dose of risedronate significantly increased BMD of the femur and vertebrae, while black cohosh extract had no significant effect on BMD individually and minimal effects upon coadministration with risedronate. Under these experimental conditions, black cohosh extract alone had no effect on BMD, nor did it negatively impact the BMD-enhancing properties of risedronate.

## 1 Introduction

Dietary supplements are often viewed as a safe alternative for the prevention and treatment of disease, frequently leading to their usage being under-reported to physicians. Combining dietary supplements with prescription medications, however, may alter the efficacy, and even safety, of a medication. The present study was designed to evaluate the potential pharmacodynamic interactions of black cohosh extract, a dietary supplement marketed to relieve the vasomotor symptoms of menopause, and the FDA-approved osteoporosis drug risedronate, prescribed to post-menopausal women to improve bone health.

Black cohosh extract is made from the roots and rhizomes of *Actaea racemosa* L. (synonym *Cimicifuga racemosa* (L.) Nutt), a perennial plant native to North America (Betz et al., 2009). Dietary supplements containing black cohosh extract are available in a variety of forms (dried extracts, liquid extracts, dried whole herb) and vary widely in their chemical composition, with some standardized on the ratio of herbal drug to native extract and others to total triterpene glycoside content (National Institutes of Health, 2023). While there are no data available on the specific number of individuals who take black cohosh extracts, it was the 20th ranked top-selling herbal supplement in 2021 (Smith et al., 2022). Black cohosh has a long history of use for women’s reproductive health (Foster, 1999), with numerous clinical studies investigating whether it can alleviate the vasomotor symptoms of menopause (Nappi et al., 2005; Osmers et al., 2005; Newton et al., 2006; Wuttke et al., 2006; Bai et al., 2007; Geller et al., 2009; Leach and Moore, 2012; Franco et al., 2016). While some studies indicated a positive effect (lessening of symptoms), others indicated worsening of symptoms or shown no benefit over placebo. The inconsistent reports on the efficacy of black cohosh products may be due to the variability of the test article used, as few include characterization or standardization (Swanson, 2002). Adulteration of black cohosh products with related species of *Actaea* has also been documented (Foster, 2013).

In addition to providing relief from the vasomotor symptoms of menopause, extracts of black cohosh have also been reported to protect against postmenopausal bone loss. Qui and others demonstrated that 25-acetylcimigenol xylopyranoside (ACCX), a component isolated from black cohosh, inhibited receptor activator of nuclear factor kappa B ligand (RANKL)-induced osteoclast differentiation of mouse bone marrow macrophages (Qiu et al., 2007). Black cohosh extract has also been associated with decreased bone loss in the ovariectomized (OVX) rat model, as well as increased bone mineral density (BMD) and enhanced callus formation in a OVX rat tibia fracture healing model (Nisslein and Freudenstein, 2003; Kolios et al., 2010; Seidlová-Wuttke et al., 2012).

Risedronate sodium is one of several bisphosphonates approved by the U.S. FDA for the treatment and prevention of osteoporosis in postmenopausal women and in 2012 was the leading branded oral bisphosphonate on the market in the U.S. (U.S. Securities and Exchange Commission, 2012). Bisphosphonates prevent bone loss by binding hydroxyapatite and inhibiting the bone resorbing action of osteoclasts through inhibition of RANKL (Yasuda et al., 1998). Nitrogen-containing bisphosphonates, such as risedronate, also block osteoclast activity by inhibiting the enzyme farnesyl pyrophosphate synthase (FPPS) (Kavanagh et al., 2006; Russell, 2011). It is possible that black cohosh extract and risedronate both protect against bone loss; risedronate via binding hydroxyapatite and inhibition of FPPS, and risedronate and black cohosh extract via inhibition of RANKL-mediated osteoclast differentiation. However, it is not known whether there are pharmacodynamic interactions when taken together that could impact the efficacy of FDA-approved bisphosphonates.

Here we investigated the individual and combined effects of black cohosh extract and risedronate at high and low doses, as well as ethinyl estradiol as a positive control, on BMD in the OVX rat, an established model of postmenopausal osteoporosis (Kalu, 1991). Risedronate treatment increased BMD compared to the OVX-vehicle controls, while black cohosh extract had no effect on BMD when administered individually. When co-administered with risedronate, black cohosh extract had some positive effects; however, BMD increases were not significantly different from animals administered risedronate alone. Taken together, the results suggest that black cohosh extract did not inhibit the effectiveness of risedronate.

## 2 Materials and methods

### 2.1 Test compounds

Risedronate sodium (Cat. No. SML0650), ethinyl estradiol (Cat No. E4876) and carboxymethylcellulose (CMC; Cat. No. C4888) were purchased from Sigma-Aldrich (St. Louis, MO). A standardized black cohosh dry extract (Cat. No. 398014; USA sourced, water/ethanol extraction; total triterpene glycoside content, 2.7%) was obtained from Euromed USA, Inc. (Presto, PA) and was considered a representative market sample. Detailed methods for test compound characterization/verification and dose certification are provided in the Supplementary Data S1.

### 2.2 Animals and experimental design

Animal procedures were approved by the NCTR Institutional Animal Care and Use Committee and followed the guidelines set forth in the Care and Use of Laboratory Animals (National Research Council, 2011). Animal rooms were maintained at 23°C ± 3°C with a relative humidity of 50% ± 20% and a 12-h light/dark cycle. Animals were housed in solid-bottom polysulfone cages with microisolator tops. Millipore-filtered tap water was provided in glass bottles with silicone stoppers. Animals were maintained on 5K96 verified casein diet 10 IF (LabDiet, St. Louis, MO), to minimize background exposure to phytoestrogens; detailed analysis of isoflavone measurements are provided in the Supplementary Data S1. Food and water were provided *ad libitum*.

**FIGURE 1:**
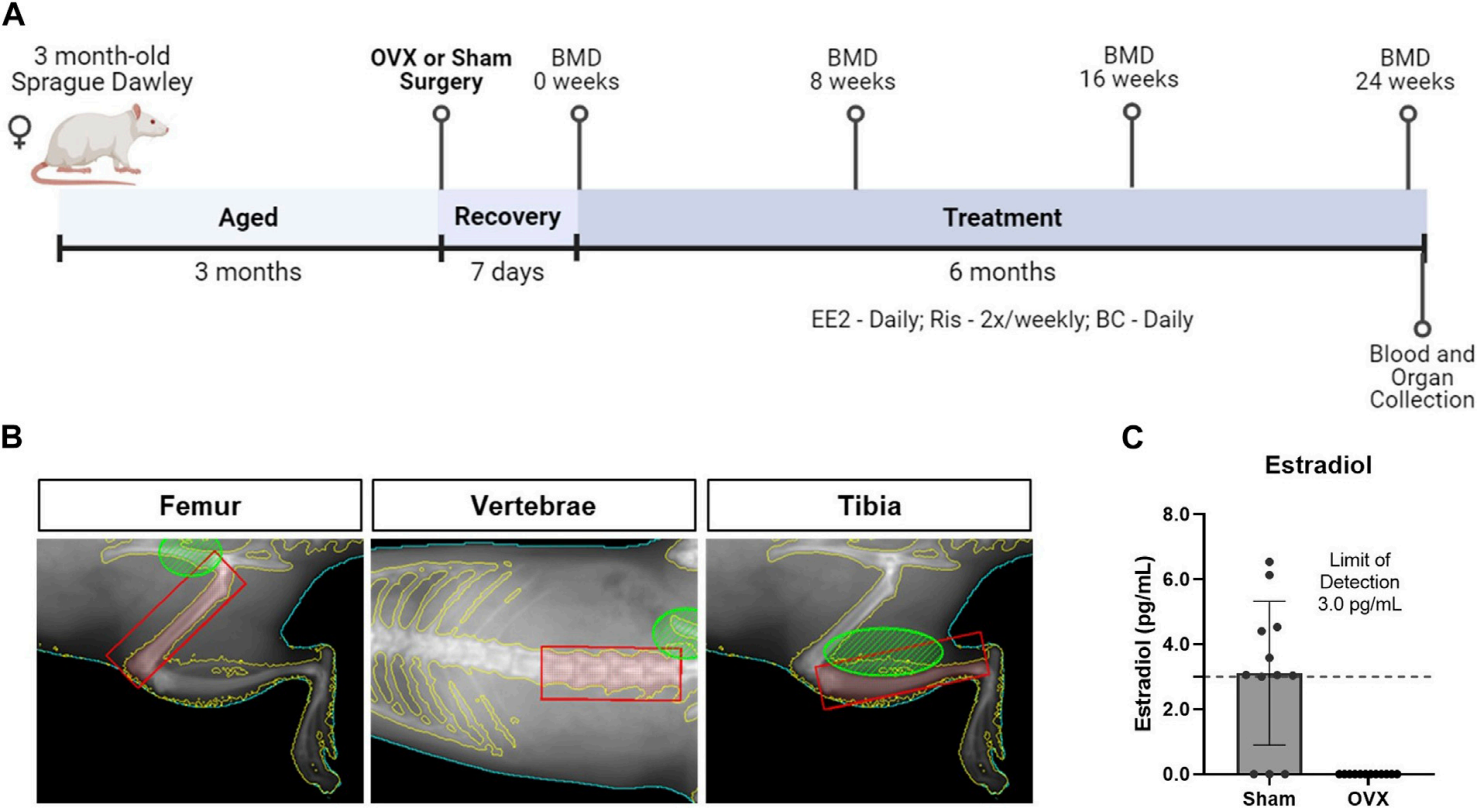
Study design to evaluate changes in bone quality in the ovariectomized rat, an established model of postmenopausal osteoporosis. **(A)** At 6 months of age, female Sprague Dawley rats underwent sham or bilateral ovariectomy (OVX). Following a 7-day recovery period, animals were treated with vehicle or test articles for 24 weeks. **(B)** Representative DEXA images show regions of interest in shaded red boxes used to quantify bone mineral density (BMD) for the femur, lumbar vertebrae (L1-L4), and tibia. All images depicted are from the same rat. **(C)** Estradiol levels, measured by ELISA, were significantly greater in sham animals compared to OVX-vehicle animals (*p* < 0.0001, n = 12). The lower limit of detection in the assay was 3 pg/mL (dashed line).

A total of 230 virgin female Sprague-Dawley rats were purchased from Harlan Industries (Indianapolis, IN) and delivered at approximately 3 months of age. At 6 months of age, animals were assigned to treatment groups such that the mean initial weights were comparable and underwent bilateral ovariectomy or a sham surgery. In brief, surgery was conducted under isoflurane anesthesia. Bilateral dorsal incisions were made to allow visualization of the viscera. The ovaries were located, removed by cauterization, and incisions closed with wound clips. In the sham control group, surgery was conducted as described, but ovaries were left intact. Animals recovered for 7 days before initial BMD assessment; dosing began the following day and continued for 24 weeks (Figure 1A).

Animals were euthanized at 1 year of age by over-exposure to carbon dioxide followed by exsanguination. Liver, kidney (paired), and uterine weights were obtained. OVX animals were examined to verify removal of ovarian tissue; three animals (n = 2 OVX-vehicle; n = 1 low dose black cohosh extract/high dose risedronate) were excluded from analysis, due to incomplete ovariectomy.

Four animals died during the study or were euthanized before the scheduled euthanasia date due to health concerns. One of the animals had a nephroblastoma, one had focal caseous pleuropneumonia due to a gavage accident, and the cause of morbidity in the other two animals could not be determined.

### 2.3 Dose selection and treatment groups

There was a total of 12 treatment groups with 18 animals assigned to each group (Table 1).

**TABLE 1:** Treatment groups and dosing.

	Treatment group	Compound(s) administered	Concentration	Route and Frequency of administration
1	Sham	CMC	0.5%	Oral Gavage; daily
2	Vehicle (OVX)	CMC	0.5%	Oral Gavage; daily
3	Lo EE2	Ethinyl Estradiol	2.5 μg/kg bw	Oral Gavage; daily
4	Hi EE2	Ethinyl Estradiol	15.0 μg/kg bw	Oral Gavage; daily
5	Lo Ris	Risedronate	1.5 μg/kg bw	Subcutaneous; biw
6	Hi Ris	Risedronate	5.0 μg/kg bw	Subcutaneous; biw
7	Lo BC	Black Cohosh Extract	10 mg/kg bw	Oral Gavage; daily
8	Hi BC	Black Cohosh Extract	100 mg/kg bw	Oral Gavage; daily
9	Lo BC + Lo Ris	Black Cohosh Extract	10 mg/kg bw	Oral Gavage; daily
		Risedronate	1.5 μg/kg bw	Subcutaneous; biw
10	Lo BC + Hi Ris	Black Cohosh Extract	10 mg/kg bw	Oral Gavage; daily
		Risedronate	5.0 μg/kg bw	Subcutaneous; biw
11	Hi BC + Lo Ris	Black Cohosh Extract	100 mg/kg bw	Oral Gavage; daily
		Risedronate	1.5 μg/kg bw	Subcutaneous; biw
12	Hi BC + Hi Ris	Black Cohosh Extract	100 mg/kg bw	Oral Gavage; daily
		Risedronate	5.0 μg/kg bw	Subcutaneous; biw

CMC, carboxymethylcellulose.

biw = twice a week.

Risedronate was solubilized in Millipore^®^-filtered water and the animals were dosed twice weekly (Monday and Thursday) by subcutaneous injection at 1.5 or 5 μg/kg bw, modeling previous bone pharmacology studies (Li et al., 1999; Yao et al., 2007; Cheng et al., 2009; Uyar et al., 2009; Shahnazari et al., 2010).

Dry black cohosh extract was mixed with 0.5% aqueous CMC. Animals were dosed daily by gavage at 10 or 100 mg/kg bw using an automated Hamilton Microlab^®^ pump (Hamilton Co., Reno, NV). Black cohosh dose selection was based on literature reports demonstrating a positive effect on bone with no reported toxicity (Seidlová-Wuttke et al., 2003a; Kolios et al., 2010). Additionally, the 10 mg/kg bw dose is similar to the upper end of the suggested human dose for treatment of menopausal symptoms (20–40 mg twice per day) (Reagan-Shaw et al., 2008).

Ethinyl estradiol, was solubilized in 0.3% aqueous CMC and administered daily by gavage at 2.5 or 15 μg/kg bw. Initially, doses of 10 and 100 μg/kg bw were selected, based on literature reports demonstrating that oral concentrations of 30 μg/kg bw reversed OVX-induced bone loss, with a dose as high as 100 μg/kg bw showing no evidence of toxicity (Ke et al., 1997; Picherit et al., 2000; Coelingh Bennink et al., 2008). However, after problems with solubility and toxicity (i.e., weight loss) concentrations were adjusted downward after three or four weeks of treatment.

### 2.4 Dual-energy X-ray absorptiometry

BMD of the femur, tibia, and L1-L4 lumbar vertebrae were measured by DEXA at weeks 0 (one day prior to start of dosing), 8, 16, and 24 (one day prior to euthanasia). The DEXA instrument (PIXImus, Lunar GE Medical Systems, Madison, WI) was calibrated using the manufacturer’s phantom mouse. All animals were anesthetized with isoflurane and placed on the DEXA tray in the ventral position with the limbs held splayed. Each animal was positioned to scan the right leg, then positioned to scan the spine area. Each region was scanned twice with repositioning of the animal between scans. The software’s inclusion and exclusion areas were used to outline the femur, tibia, and lumbar vertebrae regions of interest (ROI) (Figure 1B). BMD data for each ROI were analyzed by averaging the technical replicates. To limit repeated isoflurane exposure, only a subset of animals were subjected to DEXA scanning at weeks 8 and 16 (n = 10/group); all animals were scanned at weeks 0 and 24.

### 2.5 ELISA assays

For measurement of estradiol levels and serum bone markers, blood was collected at euthanasia by cardiac puncture into Vacutainer^®^ tubes (BD, Franklin Lakes, NJ). Samples were centrifuged at 3,000 *x g* for 10 min at room temperature; the serum was aliquoted and stored at −80°C until use.

Serum estradiol was measured using a mouse/rat estradiol ELISA kit (Cat. No. ES180S-100) from Calbiotech (Spring Valley, CA) per the manufacturer’s instructions. Samples were read on a Molecular Devices Spectramax M2 spectrophotometer (Sunnyvale, CA) and analyzed with Softmax Pro 5 software. The lower limit of detection (LOD) for estradiol was 3 pg/mL; samples below the LOD were set to zero for analysis.

### 2.6 Statistical analysis

Differences between groups were analyzed using GraphPad Prism Version 6.0 (GraphPad Software, Inc., LaJolla, CA). Differences between multiple treatment groups at single timepoints (body weight, organ weights, and BMD) were evaluated using one-way ANOVA followed by Sidak’s post-hoc test for multiple comparisons; comparisons included vehicle vs. all other groups, low risedronate treatment vs. low/high black cohosh extract + low risedronate treatment and high risedronate treatment vs. low/high black cohosh extract + high risedronate treatment. Difference in estradiol levels were evaluated using unpaired t-tests. *p* values less than 0.05 were considered significant. Data are presented as means ± standard deviation (SD), unless noted.

## 3 Results

### 3.1 Estradiol levels

Serum estradiol levels were quantified in a subset of animals from the sham and OVX-vehicle groups. Three of the twelve sham animals had estradiol levels below the LOD, while all twelve OVX-vehicle animals had undetectable levels (Figure 1C). The average estradiol concentration of the sham animals was significantly greater than the OVX-vehicle-treated group, which were 3.11 and 0 pg/mL, respectively.

### 3.2 Body and organ weights

**FIGURE 2:**
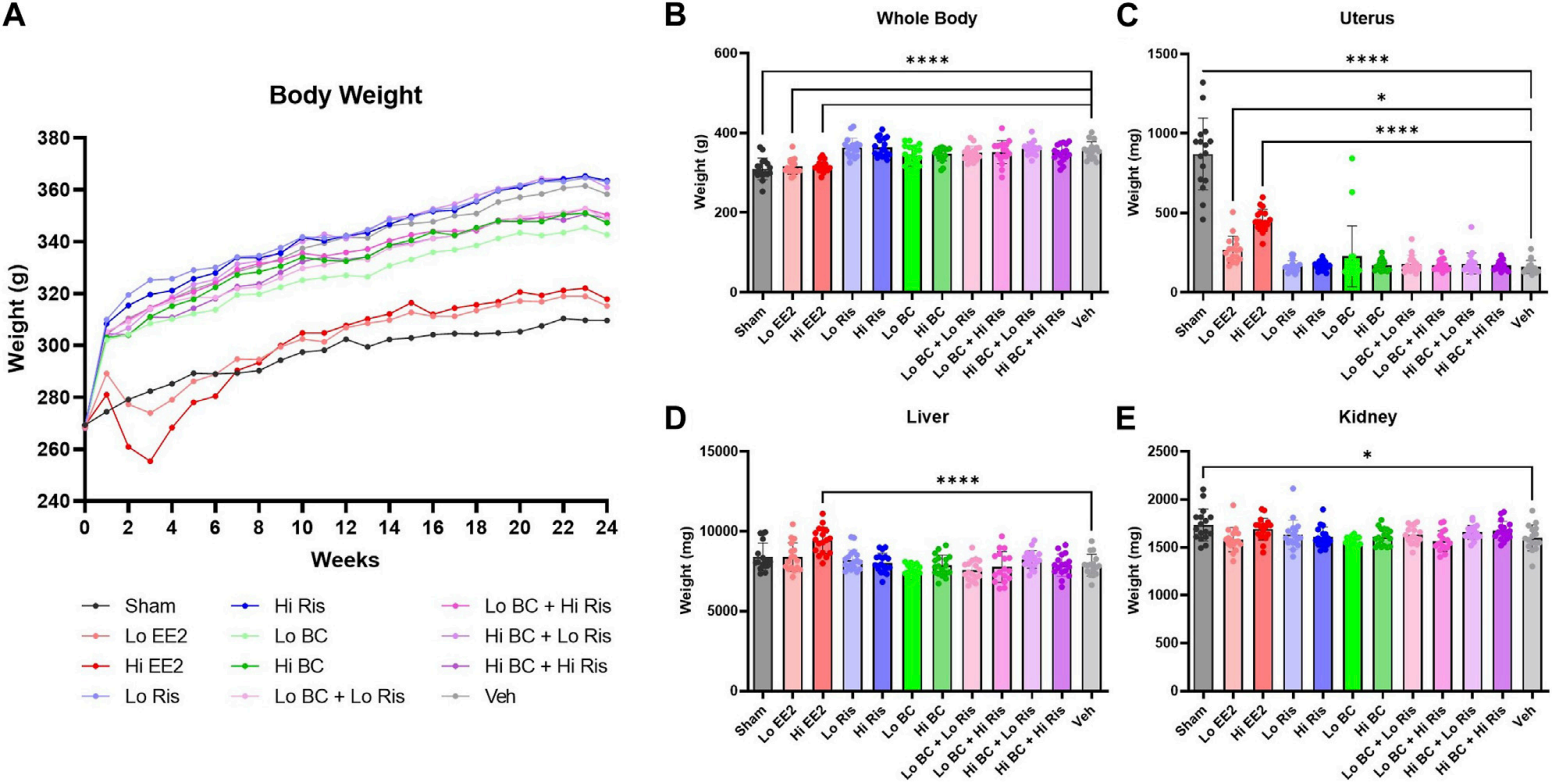
Longitudinal body, terminal body, and absolute organ weight measurements. **(A)** Mean weekly body weight measurements are plotted for each treatment group. Due to significant reductions in body weight, doses of EE2 were adjusted downward to 2.5 and 15 μg/kg body weight per day after three or four weeks of treatment for load one and load two animals. Animals in loads three and four only received the lowered doses. **(B)** Sham surgery and EE2 treatment groups had significantly lower body weight measurements at week 24, while all risedronate and black cohosh extract treatment groups showed no significant differences compared to OVX-vehicle control. **(C–E)** Uterine, liver and kidney weights at week 24 showed no significant differences associated with risedronate or black cohosh extract treatments compared to OVX-vehicle. Data plotted as mean ± SD. Significance was evaluated by one-way ANOVA with Sidak’s post-hoc for multiple comparisons. * Indicates difference vs. OVX-vehicle control; *p < 0.05, **p < 0.01, ***p < 0.001, ****p < 0.0001; n = 16–18. EE2 = ethinyl estradiol; BC = black cohosh extract; Ris = risedronate sodium; Veh = OVX-vehicle control.

Body weights of animals treated with risedronate or black cohosh extract, alone or with coadministration, did not significantly differ from OVX-vehicle controls. Estradiol and sham surgery groups had lower mean body weights than OVX-vehicle controls from week 0 through 24 (Figure 2A), with the difference reaching statistical significance at week 24 (Figure 2B).

Ovariectomy decreased uterine weights in all groups relative to the sham controls (Figure 2C). However, a dose-related increase in uterine weight was observed in the low and high ethinyl estradiol treatment groups (1.7 and 2.8-fold increase vs. OVX-vehicle, respectively). High ethinyl estradiol treatment and sham surgery groups also showed increased liver and kidney weights, respectively, at the time of sacrifice compared to OVX-vehicle controls (Figures 2D, E).

### 3.3 BMD

**FIGURE 3:**
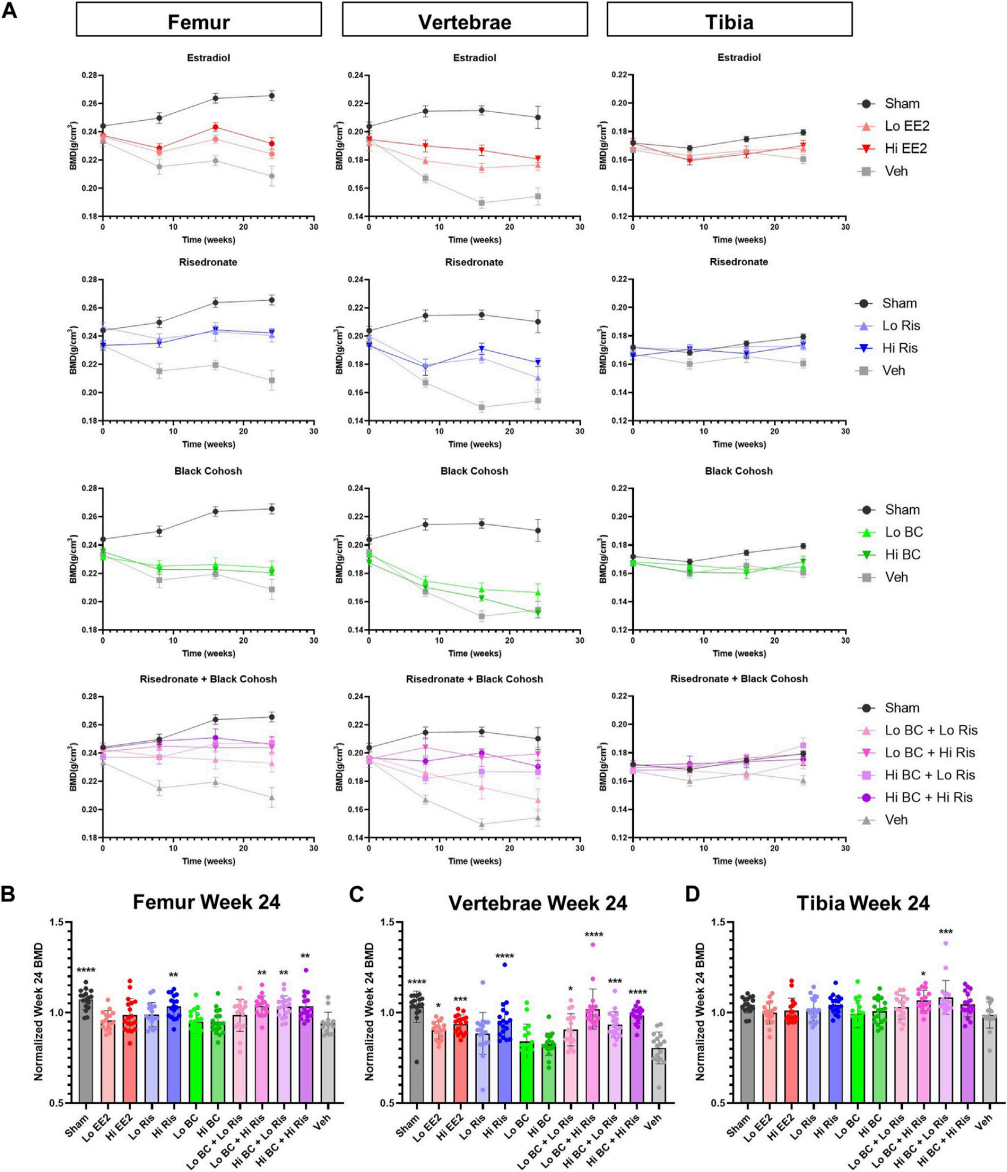
BMD of femur, vertebrae and tibia following treatment with black cohosh extract and/or risedronate. **(A)** Longitudinal measurement of BMD at 0, 8, 16, and 24 weeks of treatment is plotted to show the effects of EE2, risedronate, black cohosh extract and risedronate + black cohosh extract combination treatments, with sham and vehicle data shown on each graph. Data plotted as mean ± SEM; n = 9–10. **(B–D)** BMD at week 24 was normalized to week 0 for each animal. After normalization, significant differences in BMD were associated with Hi EE2, Hi Ris and combined BC + Ris treatments, as compared to vehicle control. No statistically significant differences were observed between Lo Ris vs. Lo/Hi BC + Lo Ris or between Hi Ris vs. Lo/Hi BC + Hi Ris groups. Data plotted as mean ± SD. Significance was evaluated by one-way ANOVA with Sidak’s post-hoc for multiple comparisons. * Indicates difference vs. OVX-vehicle control; *p < 0.05, **p < 0.01, ***p < 0.001, ****p < 0.0001; n = 14–18. EE2 = ethinyl estradiol; BC = black cohosh extract; Ris = risedronate sodium; Veh = OVX-vehicle control.

Femur, vertebrae, and tibia BMD were measured at 0, 8, 16 and 24 weeks. Mean BMD for ethinyl estradiol, risedronate, black cohosh extract and coadministration treatment groups were plotted separately to visualize trends over time (Figure 3A). Longitudinal BMD measurements revealed similar trends for femur and vertebrae BMD measurements across treatment groups, while tibia BMD trends were less apparent. In the femur and vertebrae, BMD of ethinyl estradiol (red) and risedronate (blue) groups trend higher than OVX-vehicle control but remain lower than sham surgery from 0 to 24 weeks. In contrast, treatment with black cohosh extract (green) showed minimal changes in BMD compared to OVX-vehicle control in the femur and vertebrae. Coadministration of risedronate and black cohosh extract (purple) produced modest changes in BMD of all ROIs compared to risedronate treatment alone. Net changes in tibial BMD were minimal across all control and treatment groups.

BMD at week 24 was normalized to baseline (week 0) for each animal and plotted for each ROI (Figures 3B–D). Mean BMD measurements of the femur, vertebrae, and tibia at weeks 0 and 24 are shown in Supplementary Tables S2-S4. Statistical analysis confirmed that sham surgery femurs had significantly higher BMD compared to OVX-vehicle (Figure 3B). In all high-dose risedronate treatment groups, individually and when co-administered with black cohosh extract, the BMD of the femur at week 24 was significantly greater compared to OVX-vehicle control. Black cohosh extract alone had no statistically significant effect on femur BMD, as compared to OVX-vehicle control. Interestingly, low risedronate when co-administered with high-dose black cohosh extract showed a significant increase in femur BMD as compared to OVX-vehicle control, while low risedronate treatment alone had no significant effect. However, femur BMD in this high black cohosh extract/low risedronate group was not significantly different than low-dose risedronate treatment alone (*p* = 0.6281).

Vertebral BMD followed similar trends as the femur (Figure 3C); however, the magnitude of changes in BMD were the largest among the three ROIs. The sham surgery group showed the highest mean vertebral BMD at week 24 and was 1.3-fold greater than OVX-vehicle control. Both ethinyl estradiol dose groups also had significantly higher vertebral BMDs at week 24 compared to the OVX-vehicle control. The vertebral BMD of the high-dose risedronate group was significantly larger (1.2-fold greater) than the OVX-vehicle control, whereas the low-dose risedronate treatment was statistically similar. There was no significant effect on vertebral BMD in the low or high-dose black cohosh extract groups. Low risedronate co-administered with either low or high doses of black cohosh extract led to statistically significant increases in vertebral BMD. Lastly, high-dose risedronate co-administered with low or high doses of black cohosh extract also showed significant increases in vertebral BMD, as compared to OVX-vehicle controls; however, the coadministration of black cohosh extract and risedronate did not significantly increase vertebral BMD compared to corresponding doses of risedronate alone (Lo BC + Lo Ris vs. Lo Ris, *p* = >0.9999; Hi BC + Lo Ris vs. Lo Ris, *p* = 0.7238; Lo BC + Hi Ris vs. Hi Ris, *p* = 0.2805; Hi BC + Hi Ris vs. Hi Ris, *p* = 0.9919).

Finally, normalized week 24 tibia BMD measurements showed very few statistically significant differences (Figure 3D). In the tibia, only low black cohosh extract/high risedronate and high black cohosh extract/low risedronate groups had statistically greater BMDs, as compared to OVX-vehicle control. Again, tibial BMD values in the coadministration groups were not statistically different from the corresponding doses of risedronate alone (Lo BC + Hi Ris vs. Hi Ris, *p* = 0.9945; Hi BC + Lo Ris vs. Lo Ris, *p* = 0.0588). Notably, the BMD in sham surgery tibias was not significantly different from OVX-vehicle controls (*p* = 0.2763), despite being the highest of all groups at week 24.

Changes in serum bone markers, osteocalcin, bone-specific alkaline phosphatase and C-terminal telopeptide, were also measured as markers for bone metabolism at week 24. Overall, there were no statistically significant differences between control and treatment groups (Supplementary Table S5, Figure S1).

## 4 Discussion

To ease the symptoms of menopause, many women turn to dietary supplements with little consideration of the possible unintended effects on the efficacy of their prescription medications. Here we investigated whether an extract of black cohosh had any pharmacodynamic interactions with risedronate effecting postmenopausal bone loss in the OVX rat model.

Ovariectomy decreased serum estradiol concentration, uterine weight (−81.8%), and BMD of the right femur (−6.1%), lumbar vertebrae (−19.5%) and right tibia (−1.4%) at 24 weeks, as expected. Ethinyl estradiol was included as a positive reference control to demonstrate suitability of the OVX rat model. Both low (2.5 μg/kg bw) and high doses (15 μg/kg bw) were tested; the low dose was expected to have minimal effects on bone loss and the higher dose was expected to normalize bone parameters. While the high dose did not completely normalize all biomarkers to the levels in sham animals, ethinyl estradiol treatment raised BMD levels over that of OVX-vehicle control animals in the femur, lumbar vertebrae, and tibia. Increases in the lumbar vertebrae were statistically significant. Together the data confirmed bone loss related to reductions in estrogen and appropriateness and sensitivity of the model for assessing interactions of black cohosh extract with risedronate.

Risedronate was selected for evaluation with black cohosh extract due to its prevalent use to treat osteoporosis in postmenopausal women and its demonstrated effectiveness in increasing BMD in OVX rats (Li et al., 1999; Yao et al., 2007; Cheng et al., 2009; Uyar et al., 2009; Shahnazari et al., 2010). Despite variations in dosing, previous studies were consistent in finding increased BMD, trabecular bone thickness, and volume of cortical bone area with risedronate treatment. In this study, both the low and high doses of risedronate had positive effects on BMD of the femur and the lumbar vertebrae when compared to OVX-vehicle control. As with ethinyl estradiol, BMD of the tibia was not significantly affected by risedronate treatment when compared to the OVX-vehicle group. Neither dose of risedronate influenced body weight or uterine weight, aside from the effects of ovariectomy, as expected (Erben et al., 2002).

In contrast to the positive effects observed on BMD in response to risedronate, black cohosh extract had no effect when given alone at high or low doses. After 24 weeks of treatment, BMD of the femur, lumbar vertebrae and tibia were statistically similar to OVX-vehicle control animals. Additionally, there were no differences in serum bone marker levels upon treatment with black cohosh extract. The lack of an impact on bone health upon treatment with black cohosh extract contrasts with others, which have previously reported positive associations with black cohosh extract on BMD, urine markers of bone turnover and bone morphometry (Seidlová-Wuttke et al., 2003b; Nisslein and Freudenstein, 2003; Kolios et al., 2010).

Chronic black cohosh extract administration in OVX rats also had no effect on body or uterine weights, which is consistent with previous reports demonstrating the lack of estrogenic effects of black cohosh extract. This finding was supportive of those in recent reports by Seidlová-Wuttke et al. and the National Toxicology Program, who also reported no effect of black cohosh extract on uterine weights or pubertal development in rats and mice (Seidlová-Wuttke et al., 2009; Mercado-Feliciano et al., 2012; Seidlová-Wuttke et al., 2013).

Importantly, when co-administered with risedronate, black cohosh extract did not counteract the BMD enhancing properties of risedronate. Increases in BMD were observed with certain dosage combinations but were dependent on the bone type measured. In the vertebrae, coadministration of black cohosh extract and risedronate, irrespective of the dose, had positive effects on BMD. Interestingly, the addition of a high dose of black cohosh extract with the low dose of risedronate produced a significant increase in BMD compared to vehicle in the femur and tibia, while BMD of animals administered a low dose of risedronate alone was not significantly different from vehicle. This modest increase may suggest that black cohosh extract enhances the bone-protective effects of low doses of risedronate. However, this difference was not statistically significant when comparing the coadministration groups with the same dose of risedronate treatment alone.

A consistent finding of this study was the varying patterns of BMD response across specific bones measured. Specifically, we found the greatest effect of OVX-induced bone loss and treatment-related recovery in the vertebrae, then the femur, and minimal changes in the tibia. It is known that postmenopausal bone loss in humans and in the OVX rat model, is primarily attributed to resorption of trabecular bone rather than cortical bone (Laib et al., 2001; Shin et al., 2012). Therefore, bones with higher composition of trabecular bone, such as the vertebrae, will exhibit increased rates of bone remodeling compared to those composed of more cortical bone, such as the diaphysis of long bones (Thompson et al., 1995; Shin et al., 2012). In addition to composition, bone size and mechanical loading will influence rates of remodeling, which explains the larger changes observed in the femur versus tibial BMD.

While changes in bone strength (i.e., mass, stiffness) or quality cannot be ruled out, as they were not evaluated in this study, BMD is generally regarded as a good predictor of fracture risk and bone health (Uyar et al., 2009). Further, DEXA scanning and measurement of serum biomarkers are common, non-invasive and clinically relevant analyses for osteoporosis screening in postmenopausal women. It is important to note that, although the rat has been useful for predicting effects in humans, differences in human and rat bone physiology exist, which may limit the translation of the effects observed to postmenopausal women. For example, unlike humans, rats do not develop spontaneous bone fractures in response to declining estrogen levels. Additionally, the bones of rats do not stop growing and lack a well-developed Haversian-based remodeling system that occurs in human cortical bones (Lelovas et al., 2008).

Importantly, due to the lack of characterization and/or standardization for many preparations of black cohosh, the results and conclusions drawn from this study are limited to the specific water/ethanol extract of black cohosh used and the dose regimen described. The extraction method has been shown to play a critical role in the chemical profile and biological activity of black cohosh extract (Jiang et al., 2008). While our study utilized a water/ethanol extract, many of the previous studies in the OVX rat model used an iso-propanolic extract. Comparison of extraction methods have found that the ethanolic and iso-propanolic extracts are quantitatively different in their triterpene glycoside and polyphenolic composition (Jiang et al., 2008) and thus, may explain some of the differences observed in BMD between studies. The age of the animal at ovariectomy may also have affected the observed BMD response upon treatment with black cohosh extract. Ovariectomy in this study was performed when the animals were 6 months of age, opposed to 3 months of age in previous studies (Seidlová-Wuttke et al., 2003b; Nisslein and Freudenstein, 2003; Kolios et al., 2010). Yousefzadeh and others have suggested that for osteoporosis research the preferred age for ovariectomy in rats is 6–9 months old (Yousefzadeh et al., 2020). Animals in this age-range have fewer confounding effects of age-related changes in bone growth.

The present study demonstrates that alone the water/ethanol standardized extract of black cohosh tested did not affect BMD or serum bone biomarker levels in an OVX rat model for osteoporosis. When given in combination with risedronate, black cohosh extract did not negatively impact the positive BMD-enhancing properties of the oral bisphosphonate. While increases in BMD was observed in select combinations and bone regions, the increases were not significantly different than those of risedronate alone. Taken together, there does not appear to be any pharmacodynamic synergistic effects of the FDA-approved drug risedronate and the dietary supplement black cohosh.

## Data Availability

The original contributions presented in the study are included in the article/Supplementary Material, further inquiries can be directed to the corresponding author.

## References

[B1] Bai W. Henneicke-von Zepelin H. H. Wang S. Zheng S. Liu J. Zhang Z. (2007). Efficacy and tolerability of a medicinal product containing an isopropanolic black cohosh extract in Chinese women with menopausal symptoms: a randomized, double blind, parallel-controlled study versus tibolone. Maturitas 58 (1), 31–41. 10.1016/j.maturitas.2007.04.009 17587516

[B2] Betz J. M. Anderson L. Avigan M. I. Barnes J. Farnsworth N. R. Gerdén B. (2009). Black cohosh: considerations of safety and benefit. Nutr. Today 44 (4), 155–162. 10.1097/nt.0b013e3181af63f9

[B3] Cheng Z. Yao W. Zimmermann E. A. Busse C. Ritchie R. O. Lane N. E. (2009). Prolonged treatments with antiresorptive agents and PTH have different effects on bone strength and the degree of mineralization in old estrogen‐deficient osteoporotic rats. J. Bone Min. Res. 24 (2), 209–220. 10.1359/jbmr.81005 PMC3276355 18847326

[B4] Coelingh Bennink H. J. T. Heegaard A. M. Visser M. Holinka C. F. Christiansen C. (2008). Oral bioavailability and bone-sparing effects of estetrol in an osteoporosis model. Climacteric 11 (Suppl. 1), 2–14. 10.1080/13697130701798692 18464016

[B5] Erben R. G. Mosekilde L. Thomsen J. S. Weber K. Stahr K. Leyshon A. (2002). Prevention of bone loss in ovariectomized rats by combined treatment with risedronate and 1alpha,25-dihydroxyvitamin D3. J. Bone Min. Res. 17 (8), 1498–1511. 10.1359/jbmr.2002.17.8.1498 12162504

[B6] Foster S. (1999). Black cohosh: a literature review. HerbalGram 45, 35–50.

[B7] Foster S. (2013). Exploring the peripatetic maze of black cohosh adulteration: a review of the nomenclature, distribution, chemistry, market status, analytical methods, and safety. HerbalGram 98, 32–51.

[B8] Franco O. H. Chowdhury R. Troup J. Voortman T. Kunutsor S. Kavousi M. (2016). Use of plant-based therapies and menopausal symptoms: a systematic review and meta-analysis. JAMA 315 (23), 2554–2563. 10.1001/jama.2016.8012 27327802

[B9] Geller S. E. Shulman L. P. van Breemen R. B. Banuvar S. Zhou Y. Epstein G. (2009). Safety and efficacy of black cohosh and red clover for the management of vasomotor symptoms: a randomized controlled trial. Menopause 16 (6), 1156–1166. 10.1097/gme.0b013e3181ace49b 19609225 PMC2783540

[B10] Jiang B. Reynertson K. A. Keller A. C. Einbond L. S. Bemis D. L. Weinstein I. B. (2008). Extraction methods play a critical role in chemical profile and biological activities of black cohosh. Nat. Prod. Commun. 3 (9), 1934578X0800300. 10.1177/1934578X0800300925

[B11] Kalu D. N. (1991). The ovariectomized rat model of postmenopausal bone loss. Bone Min. 15 (3), 175–191. 10.1016/0169-6009(91)90124-i 1773131

[B12] Kavanagh K. L. Guo K. Dunford J. E. Wu X. Knapp S. Ebetino F. H. (2006). The molecular mechanism of nitrogen-containing bisphosphonates as antiosteoporosis drugs. Proc. Natl. Acad. Sci. U. S. A. 103 (20), 7829–7834. 10.1073/pnas.0601643103 16684881 PMC1472530

[B13] Ke H. Z. Chen H. K. Simmons H. A. Qi H. Crawford D. T. Pirie C. M. (1997). Comparative effects of droloxifene, tamoxifen, and estrogen on bone, serum cholesterol, and uterine histology in the ovariectomized rat model. Bone 20 (1), 31–39. 10.1016/s8756-3282(96)00313-4 8988345

[B14] Kolios L. Schumann J. Sehmisch S. Rack T. Tezval M. Seidlová-Wuttke D. (2010). Effects of black cohosh (Cimicifuga racemosa) and estrogen on metaphyseal fracture healing in the early stage of osteoporosis in ovariectomized rats. Planta Med. 76 (09), 850–857. 10.1055/s-0029-1240798 20104444

[B15] Laib A. Kumer J. L. Majumdar S. Lane N. E. (2001). The temporal changes of trabecular architecture in ovariectomized rats assessed by MicroCT. Osteoporos. Int. 12, 936–941. 10.1007/s001980170022 11804020

[B16] Leach M. J. Moore V. (2012). Black cohosh (Cimicifuga spp.) for menopausal symptoms. Cochrane Database Syst. Rev. 2012 (9), CD007244. 10.1002/14651858.CD007244.pub2 22972105 PMC6599854

[B17] Lelovas P. P. Xanthos T. T. Thoma S. E. Lyritis G. P. Dontas I. A. (2008). The laboratory rat as an animal model for osteoporosis research. Comp. Med. 58 (5), 424–430. 19004367 PMC2707131

[B18] Li Q. N. Liang N. C. Huang L. F. Wu T. Hu B. Mo L. E. (1999). Skeletal effects of constant and terminated use of risedronate on cortical bone in ovariectomized rats. J. Bone Min. Metab. 17 (1), 18–22. 10.1007/s007740050058 10084397

[B19] Mercado-Feliciano M. Cora M. C. Witt K. L. Granville C. A. Hejtmancik M. Fomby L. (2012). An ethanolic extract of black cohosh causes hematological changes but not estrogenic effects in female rodents. Toxicol. Appl. Pharmacol. 263 (2), 138–147. 10.1016/j.taap.2012.05.022 22687605 PMC3422379

[B20] Nappi R. E. Malavasi B. Brundu B. Facchinetti F. (2005). Efficacy of Cimicifuga racemosa on climacteric complaints: a randomized study versus low-dose transdermal estradiol. Gynecol. Endocrinol. 20 (1), 30–35. 10.1080/09513590400020922 15969244

[B21] National Institues of Health (2023). Black cohosh - fact sheet for health professionals. Available at: https://ods.od.nih.gov/factsheets/BlackCohosh-HealthProfessional/#en7 (Accessed August 30, 2023).

[B22] National Research Council (2011). Guide for the care and use of laboratory animals. eighth edition. Washington D.C.: National Academies Press.

[B23] Newton K. M. Reed S. D. LaCroix A. Z. Grothaus L. C. Ehrlich K. Guiltinan J. (2006). Treatment of vasomotor symptoms of menopause with black cohosh, multibotanicals, soy, hormone therapy, or placebo: a randomized trial. Ann. Intern. Med. 145 (12), 869–879. 10.7326/0003-4819-145-12-200612190-00003 17179056

[B24] Nisslein T. Freudenstein J. (2003). Effects of an isopropanolic extract of Cimicifuga racemosa on urinary crosslinks and other parameters of bone quality in an ovariectomized rat model of osteoporosis. J. Bone Min. Metab. 21 (6), 370–376. 10.1007/s00774-003-0431-9 14586793

[B25] Osmers R. Friede M. Liske E. Schnitker J. Freudenstein J. Henneicke-von Zepelin H. H. (2005). Efficacy and safety of isopropanolic black cohosh extract for climacteric symptoms. Obstet. Gynecol. 105 (5), 1074–1083. 10.1097/01.AOG.0000158865.98070.89 15863547

[B26] Picherit C. Coxam V. Bennetau-Pelissero C. Kati-Coulibaly S. Davicco M. J. Lebecque P. (2000). Daidzein is more efficient than genistein in preventing ovariectomy-induced bone loss in rats. J. Nutr. 130 (7), 1675–1681. 10.1093/jn.130.7.1675 10867035

[B27] Qiu S. X. Dan C. Ding L. S. Peng S. Chen S. N. Farnsworth N. R. (2007). A triterpene glycoside from black cohosh that inhibits osteoclastogenesis by modulating RANKL and TNFalpha signaling pathways. Chem. Biol. 14 (7), 860–869. 10.1016/j.chembiol.2007.06.010 17656322

[B28] Reagan‐Shaw S. Nihal M. Ahmad N. (2008). Dose translation from animal to human studies revisited. FASEB J. 22 (3), 659–661. 10.1096/fj.07-9574LSF 17942826

[B29] Russell R. G. G. (2011). Bisphosphonates: the first 40 years. Bone 49 (1), 2–19. 10.1016/j.bone.2011.04.022 21555003

[B30] Seidlová-Wuttke D. Hesse O. Jarry H. Christoffel V. Spengler B. Becker T. (2003a). Evidence for selective estrogen receptor modulator activity in a black cohosh (Cimicifuga racemosa) extract: comparison with estradiol-17beta. Eur. J. Endorinol. 149 (4), 351–362. 10.1530/eje.0.1490351 14514351

[B31] Seidlová-Wuttke D. Jarry H. Becker T. Christoffel V. Wuttke W. (2003b). Pharmacology of Cimicifuga racemosa extract BNO 1055 in rats: bone, fat and uterus. Maturitas 44 (Suppl. 1), S39–S50. 10.1016/s0378-5122(02)00347-x 12609558

[B32] Seidlová-Wuttke D. Jarry H. Wuttke W. (2009). Effects of estradiol benzoate, raloxifen and an ethanolic extract of Cimicifuga racemosa in nonclassical estrogen regulated organs of ovariectomized rats. Planta Med. 75 (12), 1279–1285. 10.1055/s-0029-1185561 19350480

[B33] Seidlová-Wuttke D. Jarry H. Wuttke W. (2013). Plant derived alternatives for hormone replacement therapy (HRT). Horm. Mol. Biol. Clin. Investig. 16 (1), 35–45. 10.1515/hmbci-2013.0024 25436745

[B34] Seidlová-Wuttke D. Stecher G. Kammann M. Haunschild J. Eder N. Stahnke V. (2012). Osteoprotective effects of Cimicifuga racemosa and its triterpene-saponins are responsible for reduction of bone marrow fat. Phytomedicine 19 (10), 855–860. 10.1016/j.phymed.2012.05.002 22739411

[B35] Shahnazari M. Yao W. Dai W. Wang B. Ionova-Martin S. S. Ritchie R. O. (2010). Higher doses of bisphosphonates further improve bone mass, architecture, and strength but not the tissue material properties in aged rats. Bone 46 (5), 1267–1274. 10.1016/j.bone.2009.11.019 19931661 PMC3003226

[B36] Shin Y. H. Cho D. C. Yu S. H. Kim K. T. Cho H. J. Sung J. K. (2012). Histomorphometric analysis of the spine and femur in ovariectomized rats using micro-computed tomographic scan. J. Korean Neurosurg. Soc. 52 (1), 1–6. 10.3340/jkns.2012.52.1.1 22993670 PMC3440496

[B37] Smith T. Resetar H. Morton C. (2022). US sales of herbal supplements increased by 9.7% in 2021. HerbalGram 136, 42–69.

[B38] Swanson C. A. (2002). Suggested guidelines for articles about botanical dietary supplements. Am. J. Clin. Nutr. 75 (1), 8–10. 10.1093/ajcn/75.1.8 11756054

[B39] Thompson D. D. Simmons H. A. Pirie C. M. Ke H. Z. (1995). FDA Guidelines and animal models for osteoporosis. Bone 17 (4), 125S–S133. 10.1016/8756-3282(95)00285-l 8579908

[B40] U.S. Securities and Exchange Commission (2012). Warner chilcott annual report. Available at: https://www.sec.gov/Archives/edgar/data/1323854/000119312513070525/d449969d10k.htm (Accessed December 18, 2023).

[B41] Uyar Y. Baytur Y. Inceboz U. Demir B. C. Gumuser G. Ozbilgin K. (2009). Comparative effects of risedronate, atorvastatin, estrogen and SERMs on bone mass and strength in ovariectomized rats. Maturitas 63 (3), 261–267. 10.1016/j.maturitas.2009.03.018 19386450

[B42] Wuttke W. Rauš K. Gorkow C. (2006). Efficacy and tolerability of the Black cohosh (Actaea racemosa) ethanolic extract BNO 1055 on climacteric complaints: a double-blind, placebo-and conjugated estrogens-controlled study. Maturitas 55 (1), S83–S91. 10.1016/j.maturitas.2006.06.020

[B43] Yao W. Cheng Z. Koester K. J. Ager J. W. Balooch M. Pham A. (2007). The degree of bone mineralization is maintained with single intravenous bisphosphonates in aged estrogen-deficient rats and is a strong predictor of bone strength. Bone 41 (5), 804–812. 10.1016/j.bone.2007.06.021 17825637 PMC3883569

[B44] Yasuda H. Shima N. Nakagawa N. Yamaguchi K. Kinosaki M. Mochizuki S. (1998). Osteoclast differentiation factor is a ligand for osteoprotegerin/osteoclastogenesis-inhibitory factor and is identical to TRANCE/RANKL. Proc. Natl. Acad. Sci. U. S. A. 95 (7), 3597–3602. 10.1073/pnas.95.7.3597 9520411 PMC19881

[B45] Yousefzadeh N. Kashfi K. Jeddi S. Ghasemi A. (2020). Ovariectomized rat model of osteoporosis: a practical guide. EXCLI J. 19, 89–107. 10.17179/excli2019-1990 32038119 PMC7003643

