# Supplementary material for: The effect of black cohosh extract and risedronate coadministration on bone health in an ovariectomized rat model
Source: Front Pharmacol. 2024 Apr 16;15:1365151. doi: 10.3389/fphar.2024.1365151 (PMC11058223; doi:10.3389/fphar.2024.1365151)
Supplement: Supplementary file 1 [file DataSheet1.pdf]

SUPPLEMENTAL DATA

Test compound characterization and analysis of isoflavone levels in feed

Black cohosh extract solutions were prepared in 0.5% CMC at concentrations of 2 mg/mL and 20 mg/mL for analysis by liquid chromatography-tandem mass spectrometry (LC/MS/MS). Aliquots (100 µL) of each solution were transferred, while stirring, into 2 mL centrifuge tubes. Five unspiked samples for each concentration were prepared by the addition of methanol (900 µL). For preparation of spiked samples, 900 µL of methanol containing 400 ng/mL of deoxyactein (Cat. No. ASB-00004129-010; ChromaDex, Irvine, CA) was added to the 2 mg/mL dose of black cohosh extract, while 4,000 ng/mL of deoxyactein was added to the 20 mg/mL dose. The samples were then sonicated for 10 minutes at room temperature, followed by centrifugation at 21,000 x *g* for 4 minutes. For the 2 mg/mL dose, the supernatant was placed directly into 2 mL autosampler vials for analysis. For the 20 mg/mL dose, the supernatant was diluted 1:10 with methanol prior to LC/MS/MS analysis. The levels of deoxyactein in each of the black cohosh extract solutions were then determined using an Acquity I-Class ultra-performance liquid chromatography (UPLC) system (Waters Corporation, Milford, MA) coupled to a Xevo TQ-S triple quadrupole mass spectrometer (Waters Corporation) equipped with an electrospray interface operating in positive ion mode. Separation was achieved using a BEH C18 column (2.1 x 100 mm, 1.7 µm) (Waters Corporation). The column was held at 40˚C while the sample chamber was maintained at 15˚C. Mobile phase A was 15 mM ammonium acetate, while mobile phase B was acetonitrile. Gradient conditions were as follows: initial conditions at 40% B; 40-60% B in 5 minutes; 60-99% B in 0.5 minutes; hold at 99% B for 2 minutes; return to 40% B in 0.5 minutes. The total run time was 9 minutes, and the injection volume was 1 µL. All samples were injected in triplicate.

Multiple reaction monitoring (MRM) detection was used for all samples. The primary (denoted by an asterisk) and confirmatory transitions for deoxyactein are shown in Supplementary Table 1. The concentrations of black cohosh extract in the dosing formulations were certified to be within 90-110% of the nominal concentration based upon the concentration of deoxyactein in these formulations and the concentration of deoxyactein known to exist in the black cohosh extract.

Concentrations of the risedronate and ethinyl estradiol dosing solutions were verified by HPLC and UPLC analysis, respectively. All vehicle control samples were also analyzed and confirmed to be free of test article prior to use. Briefly, the samples of risedronate dosing solutions were diluted in Millipore®-filtered tap water to yield a concentration close to either of two prepared analytical standard solutions (1.5 or 5 µg/mL) and were quantified using HPLC-photo diode array (PDA) analysis (262 nm) using an Alliance HPLC system equipped with a 2996 PDA detector (Waters Corporation). Chromatographic separation was achieved with a Synergi 4µm Fusion-RP 80Å (250 x 4.6 mm) column (Phenomenex, Torrance, CA) and used an isocratic mobile phase of 5 mM phosphate buffer, pH 8.0 at 1.0 mL/min.

The samples of the ethinyl estradiol dosing solutions were prepared by taking 0.7 mL aliquots, adding 20 µL of internal standard (bisphenol A or progesterone, 50 µg/mL), followed by 70 µL saturated NaCl solution and 0.7 mL of methyl tert-butyl ether (MTBE). The sample was shaken for 10 minutes at 30 Hz. After centrifugation for 5 minutes at 14,000 rpm, the organic layer was

evaporated to dryness in a Savant Speedvac vacuum concentrator (Thermo Electron Corporation, Beverly, MA.) The residue was reconstituted in 0.1 mL of 50% methanol and quantified using an Acquity UPLC system equipped with a PDA detector (227 nm) (Waters Corporation). Chromatographic separation was achieved with a UPLC BEH C18 (2.1 x 50 mm; 1.7 µm) column (Waters Corporation) using an isocratic mobile phase of 0.1% formic acid in 50% acetonitrile at 0.8 mL/min (40˚C).

Isoflavone levels, defined as the total amount of genistein and daidzein, were determined for each lot of 5K96 verified casein diet 10 IF (Purina Mills LLC, St. Louis, MO) feed prior to use. The analytical methodology involved acid hydrolysis (20 mL of 10% hydrochloric acid in methanol) of composite diet samples (2 grams) under reflux in a Snyder distillation column for six hours or overnight incubation at room temperature. After cooling and centrifugation, an aliquot was evaporated to near dryness, 4 mL of 0.1% formic acid was added and then extracted with equal volumes of ethyl acetate three times. The combined ethyl acetate layers were evaporated, reconstituted with acetonitrile, filtered (0.2 µm) and subjected to HPLC/MS analysis. The levels of genistein and daidzein were then determined by UPLC/MS analysis using a Acquity UPLC System coupled with a Acquity QDa Mass Detector (Waters Corporation). Daidzein and genistein were eluted on an Acquity UPLC HSS T3 column (2.1 mm × 50 mm, 1.8 µm) at 40˚C with mobile phase consisting of LC-MS grade water (A) and acetonitrile (B), both containing 0.1% formic acid, at a flow rate of 0.5 mL/min. Elution started with 5% solvent B for 0.1 minute, followed by a linear gradient elution of 5 to 60% solvent B in 4.1 minutes, returning solvent B to 5% in 0.1 minute, and maintaining for 0.7 minutes to re-equilibrate the column. The eluate was detected by mass spectrometry with an electrospray ion source operating in the positive ion mode (ESI^+) using single ion recording (SIR). The monitored (M+H)+ ions were m/z 255.0 for daidzein and m/z 271.0 for genistein. Quantitation was accomplished with the standard addition method, whereby diet samples were assayed with and without known spikes of genistein and daidzein. Based on historical data for lots of 5K96 at NCTR, the tolerance level for isoflavones was set at 20 ppm.

Serum bone marker analysis
ELISA kits were purchased from LSBio (Seattle, WA) and included assays for measurement of bone-specific alkaline phosphatase (Cat. No. LS-F21496), collagen C-terminal telopeptide (Cat. No. LS-F21648), and osteocalcin (Cat. No. LS-F22801). Assays were conducted following the manufacturer's recommendations. Samples were read on a Molecular Devices Spectramax M2 spectrophotometer (Sunnyvale, CA) and analyzed with Softmax Pro 5 software. Differences between groups were evaluated using one-way ANOVA followed by Sidak's post-hoc test for multiple comparisons with a single pooled variance.

Supplemental Table 1. Multiple Reaction Monitoring (MRM) Detection for Deoxyactein

Compound	Transition	Dwell (sec)	Cone Voltage (V)	Collision Energy (eV)
*deoxyactein	661.5 > 451.4	0.1	38	14
deoxyactein	661.5 > 95.1	0.1	38	40

Supplemental Table 2. Bone Mineral Density (BMD) of the Femur

Treatment Group	N	BMD, Week 0	BMD, Week 24	BMD Difference (Between Week 24 and Week 0)
Sham	17	0.2399 ± 0.0021	0.2569 ± 0.0029	+7.1%*
Vehicle	14	0.2345 ± 0.0028	0.2204 ± 0.0031	-6.0%
Lo EE2	15	0.2381 ± 0.0028	0.2275 ± 0.0027	-4.5%
Hi EE2	18	0.2374 ± 0.0040	0.2328 ± 0.0027	-1.9%
Lo Ris	17	0.2396 ± 0.0031	0.2365 ± 0.0032	-1.3%
Hi Ris	18	0.2336 ± 0.0036	0.2412 ± 0.0034	+3.3%*
Lo BC	18	0.2347 ± 0.0028	0.2226 ± 0.0030	-5.2%
Hi BC	18	0.2335 ± 0.0028	0.2205 ± 0.0017	-5.6%
Lo BC + Lo Ris	17	0.2386 ± 0.0030	0.2345 ± 0.0042	-1.7%
Lo BC + Hi Ris	17	0.2400 ± 0.0029	0.2484 ± 0.0027	+3.5%*
Hi BC + Lo Ris	18	0.2372 ± 0.0027	0.2444 ± 0.0030	+3.0%*
Hi BC + Hi Ris	16	0.2407 ± 0.0033	0.2485 ± 0.0034	+3.2%*

BMD expressed as g/cm^2. Data are shown as mean ± SEM. $p < 0.05$.
Lo = low dose; Hi = high dose; EE2 = ethinyl estradiol; Ris = risedronate; BC = black cohosh extract.

* Significantly different when compared to vehicle

Supplemental Table 3. Bone Mineral Density (BMD) of the Lumbar Vertebrae

Treatment Group	N	BMD, Week 0	BMD, Week 24	BMD Difference (Between Week 24 and Week 0)
Sham	17	0.2004 ± 0.0023	0.2067 ± 0.0046	+3.1%*
Vehicle	16	0.1970 ± 0.0020	0.1586 ± 0.0045	-19.5%
Lo EE2	17	0.1944 ± 0.0032	0.1751 ± 0.0028	-9.9%*
Hi EE2	18	0.1940 ± 0.0027	0.1815 ± 0.0019	-6.4%*
Lo Ris	18	0.1955 ± 0.0034	0.1725 ± 0.0049	-11.8%
Hi Ris	17	0.1893 ± 0.0042	0.1793 ± 0.0027	-5.3%*
Lo BC	17	0.1955 ± 0.0029	0.1641 ± 0.0044	-16.1%
Hi BC	18	0.1904 ± 0.0023	0.1569 ± 0.0028	-17.6%
Lo BC + Lo Ris	17	0.1963 ± 0.0021	0.1775 ± 0.0041	-9.6%*
Lo BC + Hi Ris	16	0.1968 ± 0.0038	0.1995 ± 0.0033	+1.4%*
Hi BC + Lo Ris	18	0.1947 ± 0.0019	0.1818 ± 0.0037	-6.6%*
Hi BC + Hi Ris	17	0.1998 ± 0.0027	0.1966 ± 0.0026	-1.7%*

BMD expressed as g/cm^2. Data are shown as mean ± SEM. $p < 0.05$.
Lo = low dose; Hi = high dose; EE2 = ethinyl estradiol; Ris = risedronate; BC = black cohosh extract.

* Significantly different when compared to vehicle

Supplemental Table 4. Bone Mineral Density (BMD) of the Tibia

Treatment Group	N	BMD, Week 0	BMD, Week 24	BMD Difference (Between Week 24 and Week 0)
Sham	17	0.1696 ± 0.0014	0.1766 ± 0.0015	+4.1%*
Vehicle	16	0.1667 ± 0.0017	0.1645 ± 0.0031	-1.3%
Lo EE2	17	0.1691 ± 0.0020	0.1687 ± 0.0021	-0.2%
Hi EE2	17	0.1691 ± 0.0026	0.1705 ± 0.0020	+0.8%
Lo Ris	18	0.1695 ± 0.0017	0.1724 ± 0.0021	+1.7%
Hi Ris	18	0.1635 ± 0.0020	0.1705 ± 0.0025	+4.3%
Lo BC	17	0.1681 ± 0.0020	0.1665 ± 0.0019	-1.0%
Hi BC	18	0.1655 ± 0.0014	0.1668 ± 0.0025	+0.8%
Lo BC + Lo Ris	17	0.1674 ± 0.0018	0.1723 ± 0.0026	+2.9%
Lo BC + Hi Ris	17	0.1663 ± 0.0021	0.1772 ± 0.0017	+6.6%*
Hi BC + Lo Ris	18	0.1674 ± 0.0017	0.1812 ± 0.0036	+8.2%*
Hi BC + Hi Ris	17	0.1685 ± 0.0021	0.1760 ± 0.0027	+4.5%

BMD expressed as g/cm^2. Data are shown as mean ± SEM. $p < 0.05$.
Lo = low dose; Hi = high dose; EE2 = ethinyl estradiol; Ris = risedronate; BC = black cohosh extract.

* Significantly different when compared to vehicle

Supplemental Table 5. Serum Bone Markers

Treatment Group	Osteocalcin (n)	Bone-Specific Alkaline Phosphatase (n)	C-terminal Telopeptide (n)
Sham	45.83 ± 4.55 (17)	11.11 ± 0.57* (17)	6.38 ± 0.41 (17)
Vehicle	40.73 ± 4.00 (16)	8.65 ± 0.69 (16)	6.75 ± 0.45 (16)
Lo EE2	43.96 ± 3.34 (17)	9.27 ± 0.57 (17)	7.41± 0.41 (17)
Hi EE2	46.22 ± 3.71 (18)	10.25 ± 0.31 (18)	7.21 ± 0.55 (18)
Lo Ris	41.23 ± 2.48 (18)	8.39 ± 0.55 (18)	6.65 ± 0.26 (17)
Hi Ris	37.28 ± 2.27 (18)	8.46 ± 0.57 (18)	7.26 ± 0.33 (18)
Lo BC	41.02 ± 2.57 (18)	8.61 ± 0.54 (18)	6.85 ± 0.41 (18)
Hi BC	37.12 ± 2.61 (18)	8.85 ± 0.67 (18)	6.64 ± 0.27 (17)
Lo BC + Lo Ris	39.07 ± 2.52 (17)	8.14 ± 0.39 (17)	6.82± 0.48 (16)
Lo BC + Hi Ris	38.40 ± 3.97 (17)	6.99 ± 0.49 (17)	6.99 ± 0.35 (17)
Hi BC + Lo Ris	35.17 ± 3.17 (18)	7.69 ± 0.51 (18)	6.96 ± 0.43 (18)
Hi BC + Hi Ris	29.55± 2.46 (17)	8.49 ± 0.81 (17)	6.31 ± 0.48 (16)

Data presented as ng/mL. Data are shown as mean ± SEM. Number in parentheses equals number of samples analyzed. $p < 0.05$.
Lo = low dose; Hi = high dose; EE2 = ethinyl estradiol; Ris = risedronate; BC = black cohosh extract.

* Significantly different when compared to vehicle

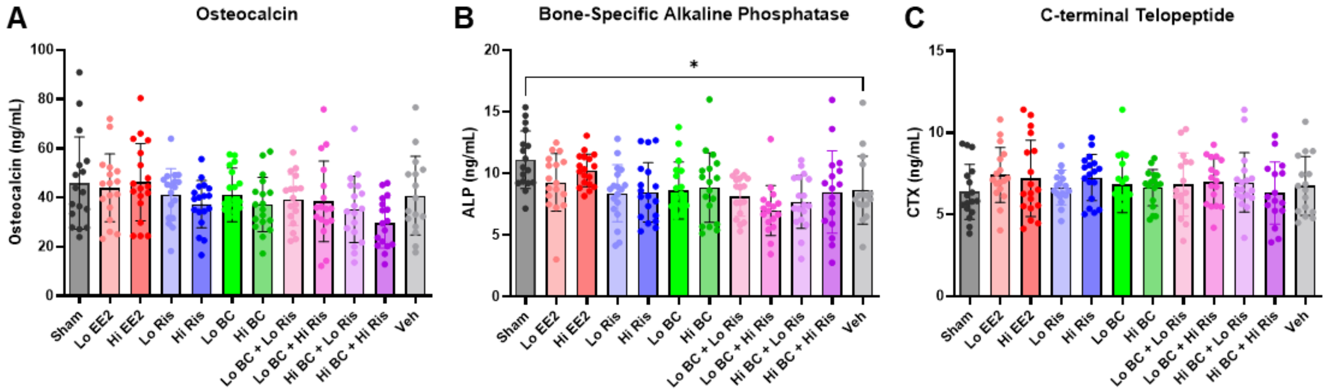

Supplemental Figure 1. Serum bone biomarker measurements were not significantly changed following black cohosh extract and/or risedronate treatment. Serum bone biomarkers **(A)** osteocalcin, **(B)** bone-specific alkaline phosphatase, and **(C)** C-terminal telopeptide were measured at week 24 at the time of sacrifice. No statistically significant differences were observed in any treatment groups, as compared to OVX-vehicle controls. Data plotted as mean ± SD. Significance was evaluated by one-way ANOVA with Sidak's post-hoc for multiple comparisons; *$p < 0.05$. EE2 = ethinyl estradiol; BC = black cohosh extract; Ris = risedronate sodium; Veh = OVX-vehicle control.
